# Trends in Costs of Care for Medicare Beneficiaries Treated in the Emergency Department From 2011 to 2016

**DOI:** 10.1001/jamanetworkopen.2020.8229

**Published:** 2020-08-06

**Authors:** Laura G. Burke, Ryan C. Burke, Stephen K. Epstein, E. John Orav, Ashish K. Jha

**Affiliations:** 1 Department of Emergency Medicine, Beth Israel Deaconess Medical Center, Boston, Massachusetts; 2 Department of Health Policy and Management, Harvard T.H. Chan School of Public Health, Boston, Massachusetts; 3 Department of Emergency Medicine, Harvard Medical School, Boston, Massachusetts; 4 Harvard Global Health Institute, Cambridge, Massachusetts; 5 Department of Biostatistics, Harvard T.H. Chan School of Public Health, Boston, Massachusetts

## Abstract

**Importance:**

There is little evidence regarding how total costs of care associated with an emergency department (ED) visit have changed, despite increasing policy focus on the value of acute care.

**Objective:**

To examine trends in total standardized 30-day costs of care associated with an ED visit.

**Design, Setting, and Participants:**

This cross-sectional study of 14 113 088 ED visits at 4730 EDs from 2011 to 2016 included a 20% national sample of traditional Medicare beneficiaries aged 65 years and older. Data analysis was conducted from August 2018 to April 2020.

**Exposures:**

Time (year) as a continuous variable.

**Main Outcomes and Measures:**

Trends in disposition from the ED and 30-day total standardized costs for all ED visits as well as the following spending components: index visit cost, physician costs, subsequent ED visit costs, subsequent inpatient costs, subsequent observation costs, non-ED outpatient care, postacute care, and aggregated total spending after the index ED visit.

**Results:**

The analytic sample consisted of 14 113 088 ED visits at 4730 EDs. The mean (SD) beneficiary age was 78.6 (8.6) years, 8 573 652 visits (60.7%) were among women, and 11 908 691 visits (84.7%) were among white patients. The proportion of patients discharged from the ED rose from 1 233 701 of 2 309 563 visits (53.4%) in 2011 to 1 279 701 of 2 268 363 visits (56.4%) in 2016. Total adjusted 30-day standardized costs of care declined from a mean (SE) of $8851 ($35.3) in 2011 to a mean (SE) of $8143 ($35.4) in 2016 (−$126/y; 95% CI, −$130 to −$121; *P* < .001) for all ED visits. This decrease was primarily associated with a decline in total spending on the index ED visit (−$48/y; 95% CI, −$50 to −$47; *P* < .001) as well as lower spending on postacute care (−$42/y; 95% CI, −$44 to −$41; *P* < .001) and subsequent inpatient care (−$34/y; 95% CI, −$36 to −$32; *P* < .001). There was an increase in spending after the index visit on downstream observation care ($3.6/y; 95% CI, $3.5 to $3.7; *P* < .001), outpatient ED care ($4.6/y; 95% CI, $4.4 to $4.8; *P* < .001), and other outpatient care ($15/y; 95% CI, $12 to $18; *P* < .001).

**Conclusions and Relevance:**

In this study, total 30-day standardized costs of ED care for Medicare beneficiaries decreased in recent years. It may be that more intensive ED spending up front is associated with reductions in total costs of an acute episode.

## Key Points

**Question:**

How have total 30-day costs of care changed over time among Medicare beneficiaries seeking care in the emergency department (ED)?

**Findings:**

In a cross-sectional study of 14 113 088 ED visits from 2011 to 2016, the mean cost of an ED visit increased among those who were discharged, but total 30-day costs of care for all ED visits decreased, largely because of a declining rate of admission to the hospital from the ED.

**Meaning:**

The findings of this study suggest that greater spending on ED care may be associated with lower total costs to Medicare.

## Teaser

This cross-sectional study examines trends in total standardized 30-day costs of care associated with an emergency department visit among Medicare beneficiaries.

## Introduction

The cost of emergency care has received substantial focus from policy makers and clinical leaders. Emergency department (ED) utilization has risen in recent years,^1^ as has the intensity of care provided in the ED, with more tests and treatments performed during a typical visit.^2,3^ While outcomes have improved for Medicare beneficiaries using the ED,^4,5^ the increased intensity of emergency care has led to greater mean charges per ED visit and greater total spending within the ED.^6^ Some have worried that increasing ED visits can trigger a cascade of downstream spending on inpatient or other types of care and have pushed to reduce ED spending, including attempting to deny payments for ED services.^7^ Operationalizing this approach without harming patients has proven difficult.^8^

While an ED visit is generally more expensive than an urgent care or office visit, it has distinct advantages, including the ability to carry out more extensive examinations and to observe patients without admitting them to the hospital. This has led some to suggest that high-intensity emergency care may increase total value by substituting for more expensive inpatient care.^2,9^ We know that while outpatient ED visit costs have risen in recent years, rates of admission from the ED have declined,^2,10^ suggesting that the emphasis on ED visit costs alone may fail to capture the overall health care savings if an expensive hospitalization is avoided.^9^ However, even in the era of alternative payment models and episodes of care, there is surprisingly little evidence regarding how total costs of an emergency care episode have changed in recent years. Such data are needed to better evaluate whether ED care is generating more value over time or whether greater up-front costs are triggering additional downstream spending and adding to the waste in the health care system.

Therefore, using national Medicare data, we sought to answer 3 questions. First, how have total 30-day costs of care associated with an ED visit changed over time? Second, what factors are associated with cost trends for ED visits? Third, have these trends varied by clinical condition?

## Methods

### Data Source

We included ED visits among a random 5% sample of traditional Medicare beneficiaries in 2009 and 2010 and a 20% sample of traditional Medicare beneficiaries in 2011 to 2016. We identified beneficiary age, race/ethnicity, sex, Medicaid eligibility, and death date from the denominator file. Beneficiary chronic conditions were identified from the Chronic Conditions Warehouse file for years 2011 to 2016 and Hierarchical Condition Categories for all years (eAppendix in the Supplement). We identified all claims within 30 and 90 days of the index ED visit for physician services, outpatient services, hospital admissions, and postacute care services (including skilled nursing and home health services).

The Office of Human Research Administration at the Harvard T.H. Chan School of Public Health approved this study. Requirement for informed consent was waived because the data were deidentified data from Medicare claims. The study is reported in accordance with the Strengthening the Reporting of Observational Studies in Epidemiology (STROBE) reporting guideline

### ED Visits

Our primary analyses used the years 2011 to 2016 because of the larger sample size and availability of Chronic Condition Warehouse categories to adjust for chronic conditions. After identifying ED visits from facility claims from January 1, 2011, to December 1, 2016, we assigned disposition to each visit as follows: admitted to the hospital, placed in observation status, transferred to another hospital, died in the ED, and discharged from the ED (eAppendix in the Supplement). The principal diagnosis was classified using the Healthcare Utilization Project Clinical Classification Software categories. The analysis was limited to the 40 most frequent diagnosis categories (14 355 661 of 18 614 363 visits [77.1%]).

### Patients

Beneficiaries aged 65 years or older who were continuously enrolled in the traditional fee-for-service model were included. We determined beneficiary age, sex, race/ethnicity (self-reported), Medicaid enrollment status, and chronic conditions.

### Outcomes

#### Total 30-Day Standardized Costs

The primary outcome was total 30-day standardized costs to Medicare^11,12,13^ beginning at the time of presentation to the ED (eAppendix in the Supplement). We included all Medicare claims beginning on the date of the ED visit to 30 days and 90 days, periods that have been previously used to attribute acute care outcomes and costs.^12,13^

#### Components of Standardized Costs

Emergency care is unique in that the ED only receives a separate facility claim for outpatient visits, while visits resulting in admission are paid under the Inpatient Prospective Payment System and do not generate a separate ED facility payment. Thus, we calculated an index ED visit cost as follows. For visits resulting in discharge from the ED, the index visit cost was calculated using outpatient ED facility claims. For visits resulting in admission, the index cost was defined by the associated inpatient diagnosis-related group payment. For visits resulting in observation, the index cost was calculated by including the outpatient ED facility claim in addition to the costs of any associated observation claims. We chose this approach because the disposition decision made by the emergency physician has substantial implications for clinical outcomes and cost. Keeping a patient in the hospital who could have gone home is associated with substantially greater costs. However, inappropriate discharge from the ED can lead to patient harm, including death.^14^ Thus, we felt it was most appropriate to attribute costs associated with this disposition decision to the index ED visit.

In addition to index ED visit costs, we calculated total 30-day and 90-day spending for physician services as well as facility-based spending that occurred after the index ED episode overall and stratified as follows: postacute care, non-ED outpatient care, subsequent inpatient care, subsequent observation care, and subsequent ED care.

### Statistical Analysis

#### Time Trends in ED Disposition

All models included an indicator for visits occurring in December (eAppendix in the Supplement). We examined trends in ED disposition given that prior work has shown a declining ED admission rate,^2,4,10^ which has implications for total spending. We calculated raw annual rates for each disposition category. We calculated adjusted trends in rates of in-ED death, admission (including visits ending in transfer), observation, and discharge from the ED using a linear probability model adjusting for visit diagnosis, hospital random effects, and beneficiary demographic characteristics and chronic conditions. We calculated this separately for each disposition category.

#### Calculating Time Trends in 30-Day Total Standardized Costs for ED Visits

We converted costs to 2016 US dollars using the Consumer Price Index to adjust for inflation. We calculated unadjusted time trends by specifying an initial linear regression model with 30-day total standardized costs as the outcome and time (year) as the exposure variable, adjusting for hospital random effects. Our subsequent model incorporated visit principal diagnosis category and beneficiary age, sex, race, and Medicaid eligibility to account for any time trends in the beneficiary demographic characteristics that may also be associated with our primary outcome. Our final model further incorporated beneficiary chronic conditions (from the Chronic Condition Warehouse) to adjust for any changes in beneficiary health status over time. We repeated these models for the individual components of standardized cost.

#### Calculating Time Trends in Costs, Defined as Mean Cost of the Index ED Visit and Total Medicare Spending, Stratified by Disposition from the ED

Given evidence that the ED admission rate is decreasing, we hypothesized that the trends in the costs of the index ED visit may differ from overall trends. Specifically, we hypothesized that the mean acuity and thus index ED visit costs would increase within the admitted, observation, and discharged disposition categories as a greater number of high-acuity patients would survive the initial ED visit and patients on the margin of acuity would shift from the admitted to the observation and discharged categories over time. Thus, we repeated our models using total spending on the index ED visit as the outcome and time (year) as the linear exposure variable, again adjusting for patient demographic characteristics and chronic conditions. To evaluate the association of changing rates of admission with total annual Medicare spending for ED care, we multiplied the mean cost per visit by the population-adjusted number of visits overall as well as stratified by disposition for each year (eAppendix in the Supplement). To examine whether time trends varied by clinical condition, we repeated our main model stratified by principal diagnosis (eAppendix in the Supplement).

#### Sensitivity Analyses

We repeated our models examining costs to 90 days. As secondary analyses, we repeated our main models incorporating hospital fixed effects. We also examined time trends, adding data from 2009 to 2010 (eAppendix in the Supplement).

A 2-sided *P* < .005 was considered statistically significant for the analyses after applying a Bonferroni correction (eAppendix in the Supplement). Data were analyzed using SAS version 9.4 (SAS Institute).

## Results

### Hospital and Patient Characteristics

After excluding 572 361 visits for missing principal diagnosis codes, there were 14 113 088 ED visits from 2011 to 2016 in our sample at 4730 hospital EDs. Key patient and hospital characteristics are presented in Table 1. The mean (SD) beneficiary age was 78.6 (8.6) years, 8 573 652 visits (60.7%) were among women, and 11 908 691 visits (84.4%) were among white patients.

**Table 1.** Patient and Hospital Characteristics for Emergency Department Visits Among Medicare Beneficiaries from 2011 to 2016

Characteristic	No. (%)		
	**2011**	**2016**	**All visits**
Emergency department visits, No.	2 309 563	2 268 363	14 113 088
Patient characteristics			
Age, mean (SD), y	78.7 (8.4)	78.2 (8.6)	78.6 (8.6)
Women	1 400 681 (60.6)	1 341 785 (59.2)	8 573 652 (60.7)
Race/ethnicity^a^			
White	1 954 247 (84.6)	1 899 885 (83.7)	11 908 591 (84.4)
Black	242 722 (10.5)	237 680 (10.5)	1 467 220 (10.4)
Hispanic	45 944 (2.0)	42 393 (1.9)	265 453 (1.9)
Other^b^	62 348 (2.7)	71 541 (3.2)	410 680 (2.9)
Medicaid eligible	545 907 (23.6)	495 826 (21.9)	3 158 462 (22.4)
Comorbidity			
Dementia	655 252 (28.4)	661 680 (29.2)	3 994 238 (28.3)
Congestive heart failure	984 203 (42.6)	905 927 (39.9)	5 750 910 (40.7)
Chronic obstructive pulmonary disease	747 296 (32.4)	723 758 (31.9)	4 457 188 (31.6)
Chronic kidney disease	887 628 (38.4)	1 101 070 (48.5)	5 906 839 (41.9)
Depression	693 005 (30.0)	749 627 (33.0)	4 423 656 (31.3)
Principal diagnosis category			
Chest pain	181 586 (7.9)	166 287 (7.3)	1 060 375 (7.5)
Other lower respiratory tract disease	153 504 (6.6)	142 777 (6.3)	935 629 (6.6)
Superficial injuries	110 130 (4.8)	106 576 (4.7)	662 910 (4.7)
Abdominal pain	112 734 (4.9)	102 553 (4.5)	670 609 (4.8)
Urinary tract infections	98 983 (4.3)	109 692 (4.8)	640 764 (4.5)
Hospital characteristics^c^			
Region			
Northeast	421 857 (18.3)	403 893 (17.8)	2 553 774 (18.1)
Midwest	550 618 (23.8)	519 248 (22.9)	3 310 059 (23.5)
South	924 322 (40.0)	929 274 (41.0)	5 760 572 (40.8)
West	367 459 (15.9)	399 141 (17.6)	2 342 986 (16.6)
Beds, No.			
≤99	446 229 (19.3)	438 581 (19.3)	2 755 569 (19.5)
100-399	1 233 336 (54.3)	1 222 431 (53.9)	7 590 981 (53.8)
≥400	584 691 (25.3)	590 544 (26.0)	3 620 841 (25.7)
Profit status			
Nonprofit	1 638 302 (70.9)	1 630 602 (71.9)	10 117 938 (71.7)
For profit	336\ 085 (14.6)	328 391 (14.5)	2 050 166 (14.5)
Government, nonfederal	289 869 (12.6)	292 563 (12.9)	1 799 287 (12.8)
Teaching status			
Major	257 507 (11.2)	269 404 (11.9)	1 612 624 (11.4)
Minor	732 706 (31.7)	725 343 (32.0)	4 492 280 (31.8)
Nonteaching	1 274 043 (55.2)	1 256 809 (55.4)	7 862 487 (55.7)
Rural hospital	172 563 (7.5)	159 616 (7.0)	1 037 889 (7.4)
Safety-net hospital	435 343 (18.8)	418 916 (18.5)	2 617 038 (18.5)

^a^ Race was unknown or missing for 4203 visits in 2011, 16 864 in 2016, and 61 144 during the total study period.

^b^ Other included beneficiaries who listed their race as other as well as those who listed their race as Asian or North American native.

^c^ For all hospital characteristics except safety-net status, there were 45 307 visits with missing characteristics in 2011, 16 807 in 2016, and 145 697 during the total study period.

### Trends in Disposition From the Emergency Department

The raw proportion of patients discharged from the ED increased from 1 233 701 of 2 309 563 visits (53.4%) in 2011 to 1 279 701 of 2 268 363 visits (56.4%) in 2016; the decrease in inpatient admission (885 324 [38.3%] in 2011 and 749 482 [33.0%] in 2016) was not offset by the rise in rates of observation (146 579 [6.3%] in 2011 and 194 235 [8.6%] in 2016) or transfer (41 736 [1.8%] in 2011 and 43 283 [1.9%] in 2016) (eTable 1 and eFigure 1 in the Supplement). Adjusted time trends are presented in eTable 2 in the Supplement.

### Trends in Total 30-Day Standardized Costs

**Figure 1.**
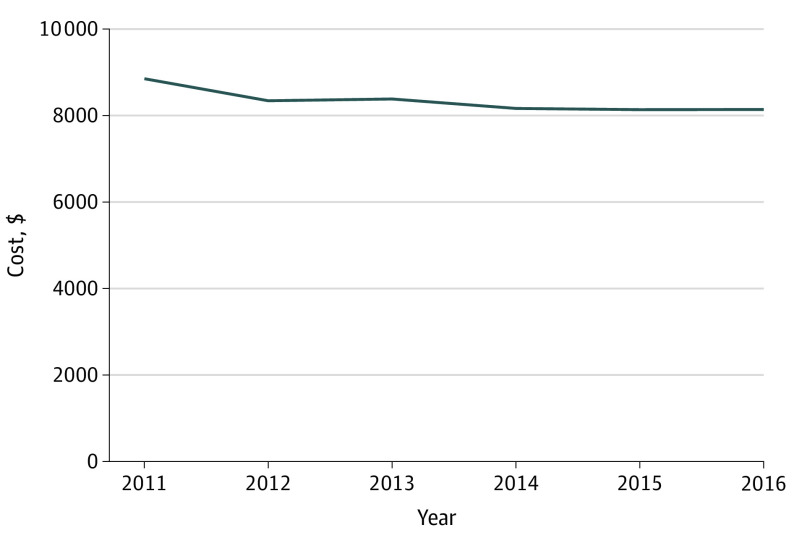
Total Adjusted Standardized Costs Within 30 Days of an Emergency Department Visit Among Medicare Beneficiaries From 2011 to 2016 Costs were converted to 2016 US dollars using the Consumer Price Index, and the model adjusts for hospital random effects, principal diagnosis, and beneficiary age, sex, Medicaid eligibility, race, and chronic conditions from the Chronic Conditions Warehouse categories. Total 30-day standardized costs include all fee-for-service claims within 30 days of an emergency department visit among continuously enrolled Medicare beneficiaries aged 65 and older presenting to a US emergency department with 1 of the 40 most frequent diagnoses.

When we examined all ED visits in aggregate, total 30-day costs of care decreased over time. Unadjusted costs are presented in eTable 3 in the Supplement. In the model adjusting only for hospital random effects and inflation, 30-day costs decreased by $99 per year (95% CI, −$104 to −$94; *P* < .001) (eTable 4 in the Supplement). The time trend was similar when adjusting for principal diagnosis as well as beneficiary age, sex, Medicaid eligibility, and race (−$86/y; 95% CI, −$91 to −$82; *P* < .001). After adjusting for chronic conditions, total adjusted 30-day standardized costs decreased by $126 per year (95% CI, −$130 to −$121; *P* < .001) from a mean (SE) of $8851 ($35.3) in 2011 to a mean (SE) of $8143 ($35.4) in 2016 (Figure 1; eTable 5 in the Supplement).

### Trends in the Components of 30-Day Standardized Costs

The adjusted cost of the index visit (all visits, not stratified by disposition) declined from a mean (SE) of $2725 ($17.2) in 2011 to a mean (SE) of $2486 ($17.2) in 2016 (−$48/y; 95% CI, −$50 to −$47; *P* < .001) (Table 2). There were increases in follow-up spending on ED care ($4.6/y; 95% CI, $4.4 to $4.8; *P* < .001), observation care ($3.6/y; 95% CI, $3.5 to $3.7; *P* < .001), and other outpatient care ($15/y; 95% CI, $12 to $18; *P* < .001) after the index ED visit, but this was offset by a reduction in spending on postacute care (−$42/y; 95% CI, −$44 to −$41; *P* < .001) and follow-up inpatient care (−$34/y; 95% CI, −$36 to −$32; *P* < .001). Thus, total adjusted spending after the index admission fell by $53 per year (95% CI, −$56 to −$49; *P* < .001), from a mean (SE) of $4865 ($33.0) in 2011 to a mean (SE) of $4504 ($33.1) in 2016. Physician spending decreased a mean of $25 per year (95% CI, −$25 to −$24; *P* < .001). Trends in cost components for all adjustment models are presented in eTable 4 in the Supplement, and mean adjusted 30-day costs by spending component are presented in eTable 5 in the Supplement.

**Table 2.** Adjusted 30-Day Standardized Costs for Emergency Department Visits Among Medicare Beneficiaries From 2011 to 2016 Overall and by Spending Component^a^

Component	Adjusted mean (SE), $		Time trend, change (95% CI), $/y	*P* value
	2011	2016		
Total 30-d costs^b^	8851 (35.3)	8143 (35.4)	−126 (−130 to −121)	<.001
Index visit costs^b^	2725 (17.2)	2486 (17.2)	−48 (−50 to −47)	<.001
Physician costs	1253 (3.2)	1144 (3.2)	−25 (−25 to −24)	<.001
Total postindex costs^c^	4865 (33.0)	4504 (33.1)	−53 (−56 to −49)	<.001
Outpatient	462 (30.5)	534 (30.5)	15 (12 to 18)	<.001
Postacute care	2520 (10.7)	2210 (10.7)	−42 (−44 to −41)	<.001
ED	105 (0.8)	119 (0.8)	4.6 (4.4 to 4.8)	<.001
Inpatient	1711 (5.9)	1554 (5.9)	−34 (−36 to −32)	<.001
Observation	19 (0.4)	41 (0.4)	3.6 (3.5 to 3.7)	<.001

Abbreviation: ED, emergency department.

^a^ Data are adjusted for inflation, hospital random effects, and principal diagnosis as well as beneficiary demographic characteristics and comorbid conditions.

^b^ All facility costs associated with the index ED visit, including any associated inpatient or observation costs if the patient remained in the hospital for further care.

^c^ All nonphysician costs occurring after the index ED visit overall and then stratified by service type.

### Trends in the Cost of the Index ED Visit, Stratified by Disposition

**Figure 2.**
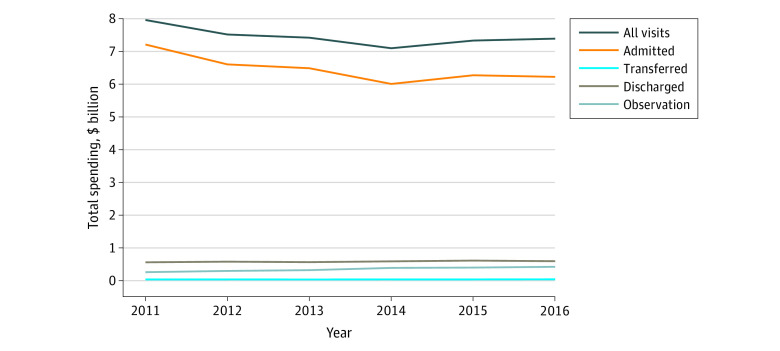
Trends in Total, Adjusted, Annual Medicare Spending on the Index Emergency Department Visit From 2011 to 2016 Among Medicare Beneficiaries for All Visits and Stratified by Disposition Total adjusted annual Medicare spending was calculated by first calculating the mean cost per visit by year from a linear regression model with index emergency department visit cost as the outcome and year as the linear exposure variable adjusting for principal visit diagnosis, and patient age, sex, Medicaid eligibility, race, and chronic conditions (from the Chronic Conditions Warehouse). Mean cost per visits were multiplied by the total number of visits in that year and adjusted for the total number of eligible beneficiaries in each year. Costs include all facility costs associated with the index ED visit, including any associated inpatient or observation costs if the patient remained in the hospital for further care.

When we examined total 30-day standardized costs for index ED visits stratified by disposition, we found that, in contrast to the decrease in costs for all ED visits, there was an increase over time within each disposition category. For example, the mean cost of the index visit for patients admitted from the ED was $7639 in 2011 vs $7726 in 2016 ($10/y; 95% CI, $6 to $14; *P* < .001) (eTable 6 in the Supplement). Among visits resulting in discharge directly from the ED, total adjusted index visit cost rose from $443 in 2011 to $456 in 2016 ($1.3/y; 95% CI, $1.1 to $1.4; *P* < .001). Total population-adjusted annual Medicare spending increased for ED visits ending in transfer, observation, and discharge (Figure 2). However, these increases were offset by the decline in total spending on visits resulting in admission. Thus, total population-adjusted annual Medicare spending on the index ED visit fell from $8.0 billion in 2011 to $7.4 billion in 2016, a 7.5% relative decrease.

### Trends in Cost by Clinical Condition

We found substantial between-condition variation in the magnitude of the relative decline in total 30-day costs (Table 3) and spending patterns (eTable 7 in the Supplement). For example, patients seen for chronic obstructive pulmonary disease saw an average relative cost decline of 2.3% (95% CI, −2.5% to −2.0%) per year compared with 0.4% (95% CI, −0.5% to −0.3%) for those with other diseases of the lower respiratory tract.

**Table 3.** Relative Decline in Total 30-Day Standardized Costs From 2011 to 2016 for Most Common Conditions Treated in the Emergency Department^a^

Condition	Visits in 2011, No. (%)	Mean (SE) standardized costs, $		Relative change from 2011-2016, % (95% CI)^b^
		2011	2016	
Chest pain	181 586 (7.9)	7359 (29)	6609 (30)	−1.5 (−1.7 to −1.4)
Other lower respiratory disease	153 504 (6.6)	12 071 (49)	11 846 (51)	−0.4 (−0.5 to −0.3)
Abdominal pain	112 734 (4.9)	8709 (43)	7836 (44)	−1.8 (−2.0 to −1.7)
Superficial injury	110 130 (4.8)	4091 (24)	3825 (24)	−1.1 (−1.3 to −0.8)
Urinary tract infection	98 983 (4.3)	8305 (38)	6957 (37)	−3.0 (−3.1 to −2.8)
Syncope	83 503 (3.6)	8669 (41)	7674 (44)	−1.9 (−2.1 to −1.8)
Pneumonia	81 674 (3.5)	13 389 (57)	11 878 (62)	−2.2 (−2.3 to −2.0)
Chronic obstructive pulmonary disease	79 202 (3.4)	8087 (43)	7197 (44)	−2.3 (−2.5 to −2.0)
Cardiac dysrhythmias	78 390 (3.4)	8800 (45)	8064 (45)	−1.4 (−1.6 to −1.0)
Back pain	70 918 (3.1)	6898 (45)	6052 (44)	−1.8 (−2.1 to −1.6)
Malaise or fatigue	66 452 (2.9)	12 086 (60)	11 696 (59)	−0.4 (−0.6 to −0.2)
Congestive heart failure	64 870 (2.8)	13 813 (69)	12 802 (73)	−1.4 (−1.6 to −1.0)
Other connective tissue disease	63 073 (2.7)	8001 (53)	7186 (54)	−1.3 (−1.5 to −1.0)
Other injuries	62 744 (2.7)	7254 (51)	7779 (64)	−0.6 (−0.9 to −0.3)
Other gastrointestinal disorders	58 805 (2.5)	7623 (47)	6924 (47)	−1.7 (−2.0 to −1.5)
Dizziness or vertigo	57 617 (2.5)	4553 (32)	4261 (32)	−0.9 (−1.2 to −0.6)
Fluid electrolyte disorders	56 640 (2.5)	10 549 (634)	9778 (635)	−1.7 (−1.6 to −1.0)
Gastrointestinal hemorrhage	53 165 (2.3)	12 528 (60)	11 717 (63)	−1.3 (1.5 to −1.0)
Other nontraumatic joint disorders	52 787 (2.3)	8631 (52)	8337 (50)	−0.4 (−0.6 to −0.1)
Sprain	52 101 (2.3)	3183 (28)	2799 (29)	−1.9 (−2.3 to −1.5)

^a^ Costs were converted to 2016 US dollars using the Consumer Price Index. Adjusted rates derived from a linear regression model with total 30-day costs and time (year) as the variable, adjusting for hospital random effects and principal diagnosis as well as beneficiary age, sex, Medicaid eligibility, race, and chronic conditions. Principal diagnosis categories were classified by Healthcare Utilization Project Single-Level Clinical Classifications Software categories.

^b^ The slope for total-day adjusted standardized costs for each condition was divided by the starting (2011) adjusted 30-day standardized costs to get the mean relative change in costs over time; 95% CIs were calculated by dividing the lower and upper confidence intervals for the slope for the trend in total 30-day standardized costs divided by the mean adjusted standardized cost in 2011.

### Sensitivity Analyses

Our findings were similar when incorporating hospital fixed effects (eTable 8 in the Supplement). The decline was even greater when extending the analysis to include 2009 and 2010 data (−$267/y; 95% CI, −$271 to −$264; *P* < .001) (eTable 9 in the Supplement). Adjusted 90-day standardized costs fell from $14 346 in 2011 to $13 266 in 2016 (−$205/y; 95% CI, −$216 to −$194; *P* < .001) (eFigure 2 and eTable 10 in the Supplement).

## Discussion

Between 2011 and 2016, total 30-day and 90-day spending associated with an ED visit decreased, largely because of declining rates of admission from the ED, although other factors played a role. While there was a small increase in spending on various types of downstream outpatient care, this increase was much smaller than the decline in spending on inpatient and postacute care. Not surprisingly, in analyses stratified by disposition, the mean cost per index visit rose within each group. However, total Medicare spending on index ED visits fell over time, as fewer beneficiaries were admitted to the hospital for costly inpatient care at the conclusion of their ED stay. The declines in total costs of care were present across nearly every major diagnosis, although the magnitude of the decline varied by condition.

Our results suggest that the focus on rising ED utilization and costs may fail to capture the full costs associated with an acute episode and the role that ED care plays in moderating these costs.^15^ While there has been understandable attention paid to the fact that an outpatient ED visit is more expensive than an office or urgent care visit,^16^ we know less about the ED’s role in total acute care spending. Because EDs can perform advanced diagnostics and treatments, they can represent a lower-cost alternative to hospitalization.^9^ Accordingly, we found that when we examined all ED visits in aggregate, the total costs of the index visit decreased over time as fewer patients were admitted to the hospital. This decline in up-front costs for ED patients did not lead to a commensurate increase in downstream spending, and on balance, total spending declined over time at 30 and 90 days. While the 8% relative decline in total 30-day costs during the 6-year study period may be considered modest, this represents substantial savings to Medicare and stands in contrast to the overall increase in health care spending more broadly during the same period.^17^ Given that mortality has also declined for Medicare beneficiaries using the ED during this period,^4^ the decline in total spending on ED episodes of care appears not to have occurred at the expense of patient outcomes. However, data using other outcome measures would be helpful to further explore this hypothesis.

Our finding that spending on all outpatient (not just ED) utilization rose while inpatient utilization declined is consistent with broader trends in health care delivery. Thus, while outpatient ED visits are often portrayed as a failure of the primary care system to manage acute and chronic diseases, our results suggest some of the rise in outpatient ED visits may actually reflect the success of the ED in avoiding more costly inpatient care. There have been a number of hypothesized mechanisms for the decline in hospital admissions in recent years, including the growth of alternative payment models.^18,19,20^ Alternatively, limited hospital capacity may have incentivized emergency physicians to discharge a greater number of patients on the margin of acuity.^21^ Regardless of the precise mechanism, our results suggest that a narrow focus on cost trends for ED visits ending in discharge (rather than all visits) may obscure the broader role of the ED in the transition to an outpatient delivery model of acute care.

Our results are consistent with prior work suggesting that greater up-front spending may be associated with less overall health care spending.^12,22^ Prior studies^2,3,10,23,24^ have shown an increase in emergency care intensity, while rates of hospitalization have declined for ED patients. Our study extends this work by examining the association of trends in ED care with overall Medicare spending. Our observed increase in outpatient ED spending alongside a reduction in total costs of care for ED patients supports the notion that health care spending should be viewed from a broader perspective^12,22^ rather than from a focus on individual service lines. Indeed, the concept of the episode of care was the basis for the recent policy shift toward the bundling of services.

### Limitations

This study has limitations. It was conducted among traditional Medicare beneficiaries aged 65 years and older, and the results may not be generalizable to other populations. However, prior studies have suggested that the decrease in admission rates from the ED has occurred across a variety of payer types and patient populations.^10^ Thus, while the magnitude of the trend may differ for beneficiaries of other payers, it is likely that the overall concept of substitution of higher intensity emergency care for more costly inpatient care still holds. Additionally, it is possible that the trends in this observational study may be due to an unmeasured decline in the severity of patients presenting to the ED over time. However, if this were the case, we would have expected to find a decline in spending for all service types, as patients with more severe illness tend to use more of all types of care. However, we found an increase in spending on all types of outpatient care, suggesting that an unmeasured decline in patient severity is unlikely to explain our findings. Furthermore, prior analyses in this population indicate that the acuity has actually risen over time.^4^

## Conclusions

In this study, total 30-day and 90-day costs of care for traditional Medicare beneficiaries visiting the ED decreased from 2011 to 2016. This trend was associated with lower use of inpatient care at the time of the index ED visit as well as lower use of inpatient and postacute care in the follow-up period. These findings suggest that the increase in spending on outpatient ED care may be associated with lower total Medicare spending.

## Notes

Supplement. **eAppendix. **Supplemental Methods **eFigure 1. **Changes in Emergency Department Disposition Among Medicare Beneficiaries Treated in the United States From 2011 to 2016 **eFigure 2. **Trends in Adjusted Total 90-Day Costs by Year for Emergency Department (ED) Visits Among Medicare Beneficiaries From 2011 to 2016 **eTable 1. **Unadjusted Rates of Disposition From the Emergency Department by Year Among Medicare Beneficiaries From 2011 to 2016 **eTable 2. **Adjusted Rates of Disposition From the Emergency Department by Year Among Medicare Beneficiaries From 2011 to 2016 **eTable 3. **Unadjusted Total 30-Day Costs and Components of Spending by Year for Emergency Department (ED) Visits Among Medicare Beneficiaries From 2011 to 2016 Without and With Adjustment for Inflation **eTable 4. **Time Trends in 30-Day Costs Associated With Emergency Department Visits From 2011 to 2016 Among Medicare Beneficiaries by Adjustment Model **eTable 5.** Adjusted Total 30-Day Costs and Components of Spending by Year for Emergency Department Visits Among Medicare Beneficiaries From 2011 to 2016 **eTable 6. **Trends in Adjusted Cost of the Index Visit From 2011 to 2016 Among Medicare Beneficiaries Using the Emergency Department, Overall and Stratified by Disposition **eTable 7. **Adjusted Total 30-Day Costs and Components of Spending by Year for Emergency Department (ED) Visits Among Medicare Beneficiaries From 2011 to 2016 for the 10 Most Frequent Conditions **eTable 8. **Comparing Time Trends in 30-Day Standardized Costs Overall and by Spending Component, Adjusted for Hospital Fixed Effects vs Random Effects **eTable 9. **AdjustedTime Trends in Total 30-Day Costs and Components of Spending by Year for Emergency Department (ED) Visits Among Medicare Beneficiaries From 2009 to 2016 **eTable 10. **Trends in Adjusted Total 90-Day Costs and Components of Spending for Emergency Department (ED) Visits Among Medicare Beneficiaries From 2011 to 2016
