# Supplementary material for: Trends in Costs of Care for Medicare Beneficiaries Treated in the Emergency Department From 2011 to 2016
Source: JAMA Netw Open. 2020 Aug 6;3(8):e208229. doi: 10.1001/jamanetworkopen.2020.8229 (PMC7411538; doi:10.1001/jamanetworkopen.2020.8229)
Supplement: Supplement. — eAppendix. Supplemental Methods eFigure 1. Changes in Emergency Department Disposition Among Medicare Beneficiaries Treated in the United States From 2011 to 2016 eFigure 2. Trends in Adjusted Total 90-Day Costs by Year for Emergency Department (ED) Visits Among Medicare Beneficiaries From 2011 to 2016 eTable 1. Unadjusted Rates of Disposition From the Emergency Department by Year Among Medicare Beneficiaries From 2011 to 2016 eTable 2. Adjusted Rates of Disposition From the Emergency Department by Year Among Medicare Beneficiaries From 2011 to 2016 eTable 3. Unadjusted Total 30-Day Costs and Components of Spending by Year for Emergency Department (ED) Visits Among Medicare Beneficiaries From 2011 to 2016 Without and With Adjustment for Inflation eTable 4. Time Trends in 30-Day Costs Associated With Emergency Department Visits From 2011 to 2016 Among Medicare Beneficiaries by Adjustment Model eTable 5. Adjusted Total 30-Day Costs and Components of Spending by Year for Emergency Department Visits Among Medicare Beneficiaries From 2011 to 2016 eTable 6. Trends in Adjusted Cost of the Index Visit From 2011 to 2016 Among Medicare Beneficiaries Using the Emergency Department, Overall and Stratified by Disposition eTable 7. Adjusted Total 30-Day Costs and Components of Spending by Year for Emergency Department (ED) Visits Among Medicare Beneficiaries From 2011 to 2016 for the 10 Most Frequent Conditions eTable 8. Comparing Time Trends in 30-Day Standardized Costs Overall and by Spending Component, Adjusted for Hospital Fixed Effects vs Random Effects eTable 9. AdjustedTime Trends in Total 30-Day Costs and Components of Spending by Year for Emergency Department (ED) Visits Among Medicare Beneficiaries From 2009 to 2016 eTable 10. Trends in Adjusted Total 90-Day Costs and Components of Spending for Emergency Department (ED) Visits Among Medicare Beneficiaries From 2011 to 2016 [file jamanetwopen-3-e208229-s001.pdf]

# Supplementary Online Content

Burke LG, Burke RC, Epstein SK, Orav EJ, Jha AK. Trends in costs of care for Medicare beneficiaries treated in the emergency department from 2011 to 2016. *JAMA Netw Open*. 2020;3(8):e208229. doi:10.1001/jamanetworkopen.2020.8229

**eAppendix.** Supplemental Methods
**eFigure 1.** Changes in Emergency Department Disposition Among Medicare Beneficiaries Treated in the United States From 2011 to 2016
**eFigure 2.** Trends in Adjusted Total 90-Day Costs by Year for Emergency Department (ED) Visits Among Medicare Beneficiaries From 2011 to 2016
**eTable 1.** Unadjusted Rates of Disposition From the Emergency Department by Year Among Medicare Beneficiaries From 2011 to 2016
**eTable 2.** Adjusted Rates of Disposition From the Emergency Department by Year Among Medicare Beneficiaries From 2011 to 2016
**eTable 3.** Unadjusted Total 30-Day Costs and Components of Spending by Year for Emergency Department (ED) Visits Among Medicare Beneficiaries From 2011 to 2016 Without and With Adjustment for Inflation
**eTable 4.** Time Trends in 30-Day Costs Associated With Emergency Department Visits From 2011 to 2016 Among Medicare Beneficiaries by Adjustment Model
**eTable 5.** Adjusted Total 30-Day Costs and Components of Spending by Year for Emergency Department Visits Among Medicare Beneficiaries From 2011 to 2016
**eTable 6.** Trends in Adjusted Cost of the Index Visit From 2011 to 2016 Among Medicare Beneficiaries Using the Emergency Department, Overall and Stratified by Disposition
**eTable 7.** Adjusted Total 30-Day Costs and Components of Spending by Year for Emergency Department (ED) Visits Among Medicare Beneficiaries From 2011 to 2016 for the 10 Most Frequent Conditions
**eTable 8.** Comparing Time Trends in 30-Day Standardized Costs Overall and by Spending Component, Adjusted for Hospital Fixed Effects vs Random Effects
**eTable 9.** Adjusted Time Trends in Total 30-Day Costs and Components of Spending by Year for Emergency Department (ED) Visits Among Medicare Beneficiaries From 2009 to 2016
**eTable 10.** Trends in Adjusted Total 90-Day Costs and Components of Spending for Emergency Department (ED) Visits Among Medicare Beneficiaries From 2011 to 2016

This supplementary material has been provided by the authors to give readers additional information about their work.

© 2020 Burke LG et al. *JAMA Network Open.*

## eAppendix. Supplemental Methods

Our primary analysis used years 2011-2016 for which we used a random 20% sample of beneficiaries. In our sensitivity analysis, we added 2009-2010, for which a 5% sample is available for outpatient visits. Thus, we used the random 5% sample of beneficiaries for all visits in those years.

Physician claims were obtained from the carrier file and outpatient claims from the outpatient file. Hospital admissions were identified in Medicare Provider Analysis and Review (MedPAR) file for 2009 and 2010 and the inpatient file for years 2011-2016; claims for post-acute care services from the inpatient, skilled nursing facility, and home health claim files; physician claims from the Carrier file; and outpatient claims from the outpatient file.

In our primary analysis using 2011-2016, we used Beneficiary Chronic Conditions Warehouse (CCW) categories from the CCW data files to adjust for chronic conditions. In our sensitivity analysis using 2009-2016, we used CMS Hierarchical Condition Categories on based on diagnoses coded on claims during the respective year. We limited the number of allowable diagnoses to 9 on all claims for all years based on prior literature (Tsugawa et al JAMA Intern Med. 2019 Jun 26.doi: 10.1001/jamainternmed.2019.1005).

**Identifying Emergency Department (ED) Visits**
ED visits were identified in the outpatient and inpatient facility claims files using revenue center codes 0450-0459 and 0981.

**Classifying ED Visit Disposition**
We created a mutually exclusive classification of disposition from the ED according to the following hierarchy:

1) Admitted (an inpatient clam with an ED revenue center code)
2) Observation (an outpatient ED visit with an associated observation claim)
3) Transferred to another hospital (outpatient ED visit with a discharge destination of transferred to another acute care hospital)
4) Died in the ED- an outpatient ED Visit without an observation claim, without a discharge disposition of transferred and with a death date on the same day as the ED Visit or a discharge status code indicating patient expired
5) Discharged- an outpatient ED visit that did not meet any of the above classifications

For the purposes of calculating adjusted time trends for each disposition category we aggregated admitted and transferred patients into a single category.

**Calculating Standardized Costs**
Standardized costs were calculated using the methodology described by the Centers for Medicare and Medicaid Services. Details for this methodology are available at:
https://www.cms.gov/Research-Statistics-Data-and-Systems/Statistics-Trends-and-Reports/Medicare-Geographic-Variation/Downloads/Geo_Var_PUF_Technical_Supplement.pdf

**Calculating Annual Total Spending on the Index Visit for All Eligible Beneficiaries**
In order to calculate total annual Medicare spending on the index ED visit, we first determined the mean adjusted cost of the index visit for all visits and multiplied by the total numbers of visits in our sample for each year. We then repeated this calculation stratified by ED visit disposition (admitted, discharged, observation, transferred). For years 2012-2016 we multiplied this value by an adjustment factor for the total number of eligible beneficiaries (number of eligible beneficiaries in 2011/number of eligible beneficiaries in that respective year). While we excluded visits in December 2016 from our primary analyses because of the lack of complete data, we included the number of visits overall and by disposition for all visits in 2016 for this total annual spending calculation to make comparable projections across years.

**Addressing claims occurring in December**
Because we did not have data for claims after December 31, 2016, we exclude visits in the final 30 days of December 2016 and then incorporated an indicator for claims occurring in December in all other data years.

**Trends in Cost by Clinical Condition**
To examine the relative change in cost for each condition, we divided the slope from our model by the mean adjusted 30-day standardized costs in 2011 to get a relative change per year over time. We calculated this relative change for the 20 most frequently treated conditions in 2011 and examined the trends in total 30-day costs and cost components for the ten most common conditions treated.

**Adjusting for Multiple Comparisons**
We adjusted our significance threshold to $p<.005$ to account for 9 comparisons of time trends in total 30-day standardized costs as well as individual components of spending.

**Centers for Medicare and Medicaid Services Chronic Conditions Warehouse (CCW) Categories**

We incorporated the following CCW chronic conditions in our adjustment model:

| | |
|---|---|
| Acquired Hypothyroidism | Chronic Kidney Disease |
| Acute Myocardial Infarction | Chronic Obstructive Pulmonary Disease |
| Alzheimer's Disease | Depression |
| Alzheimer's Disease, Related Disorders, or Senile Dementia | Diabetes |
| Anemia | Glaucoma |
| Asthma | Heart Failure |
| Atrial Fibrillation | Hip / Pelvic Fracture |
| Benign Prostatic Hyperplasia | Hyperlipidemia |
| Cancer, Colorectal | Hypertension |
| Cancer, Endometrial | Ischemic Heart Disease |
| Cancer, Breast | Osteoporosis |
| Cancer, Lung | Rheumatoid Arthritis / Osteoarthritis |
| Cancer, Prostate | Stroke / Transient Ischemic Attack |
| Cataract | |

Additional information may be obtained at https://www2.ccwdata.org/web/guest/condition-categories (Last Accessed April 2, 2020)

© 2020 Burke LG et al. *JAMA Network Open.*

**eFigure 1. Changes in Emergency Department (ED) Disposition[a] Among Medicare Beneficiaries[b] Treated in the United States From 2011 to 2016**

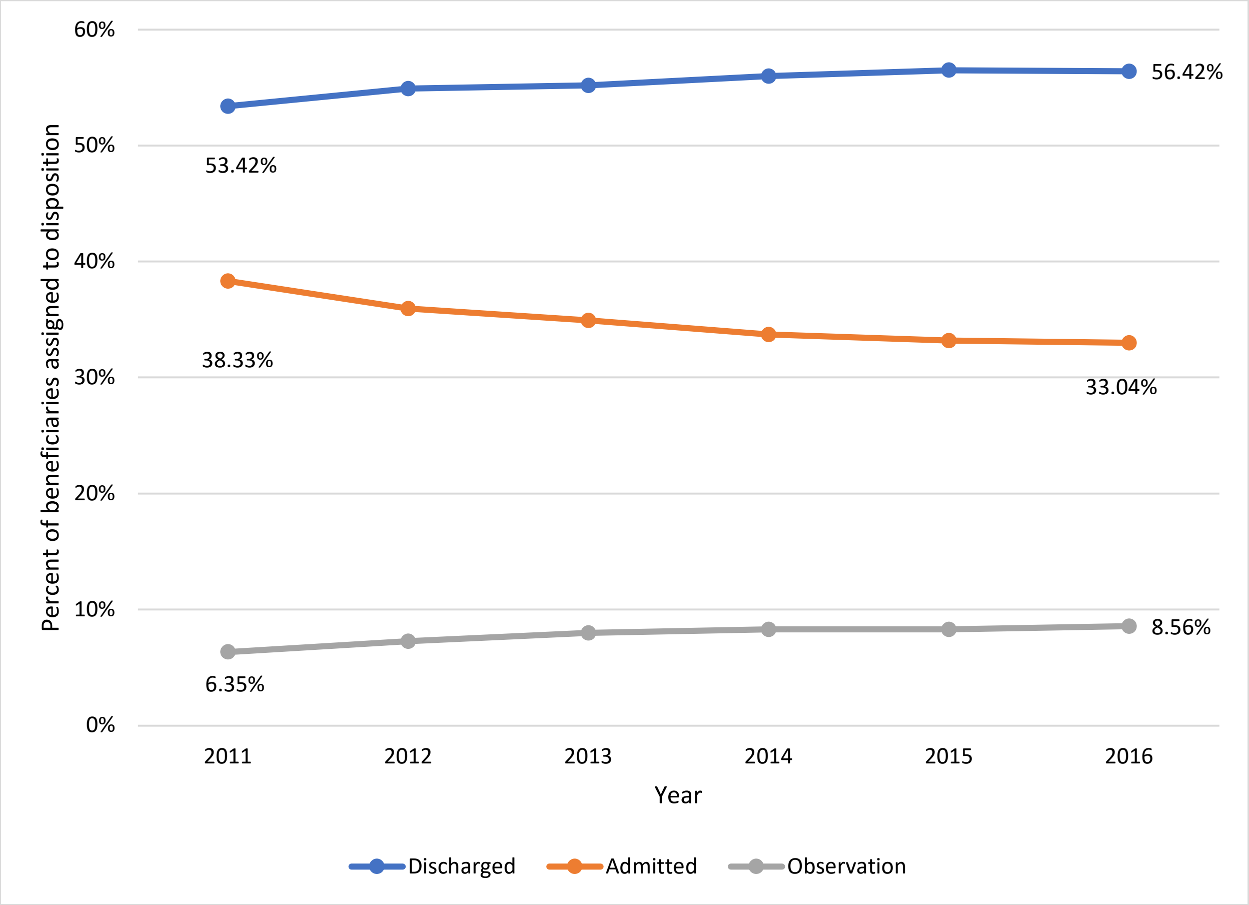


[a]Beneficiaries were assigned a disposition from the ED according the following mutually exclusive hierarchy of categories, admitted (inpatient ED visit), observation (outpatient ED visit with an associated observation claim), died in the ED (outpatient ED visit with a same day death date), discharged from the ED. [b]Beneficiaries age 65 and older seeking care in the emergency department at an acute care hospital in the 50 United States and the District of Columbia.

© 2020 Burke LG et al. *JAMA Network Open.*

**eFigure 2. Trends in Adjusted[a] Total 90-Day Costs by Year for Emergency Department (ED) Visits Among Medicare Beneficiaries From 2011 to 2016**

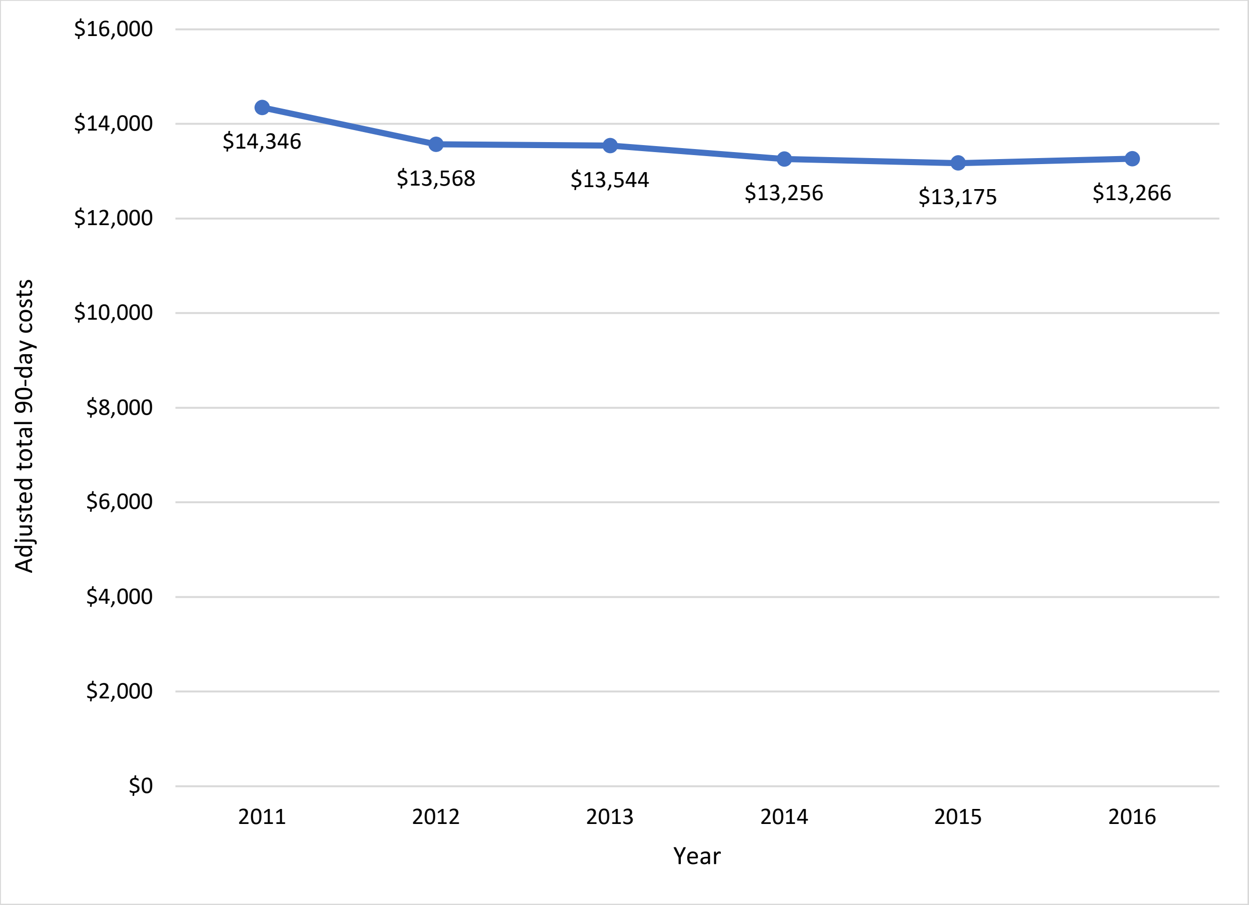


a Costs were converted to 2016 dollars using the Consumer Price Index and model adjusts for hospital random effects, principal diagnosis as well as beneficiary age, sex, Medicaid eligibility, race and chronic conditions (Chronic Conditions Warehouse categories). b Total 90-day standardized costs of all fee-for-service claims within \90 days of an ED among continuously-enrolled Medicare beneficiaries age 65 and older presenting to a US ED with one of the top 40 most frequent diagnoses.

© 2020 Burke LG et al. *JAMA Network Open.*

## eTable 1. Unadjusted Rates of Disposition[a] From the Emergency Department (ED) by Year Among Medicare Beneficiaries[b] From 2011 to 2016

| Year | Number of Visits | Visits per 1000 Beneficiaries | Admitted | Observation | Transferred | Died in the ED | Discharged |
|---|---|---|---|---|---|---|---|
| **2011** | 2,309,563 | 369 | 38.3% | 6.3% | 1.8% | 0.10% | 53.4% |
| **2012** | 2,324,908 | 366 | 36.0% | 7.3% | 1.8% | 0.10% | 54.9% |
| **2013** | 2,292,873 | 356 | 34.9% | 8.0% | 1.8% | 0.09% | 55.2% |
| **2014** | 2,337,936 | 356 | 33.7% | 8.3% | 1.9% | 0.09% | 56.0% |
| **2015** | 2,579,445 | 371 | 33.2% | 8.3% | 1.9% | 0.09% | 56.5% |
| **2016[c]** | 2,268,363 | 338 | 33.0% | 8.6% | 1.9% | 0.07% | 56.4% |

[a]A mutually exclusive hierarchy of disposition categories was created as follows: admitted (inpatient claim associated with the ED visit), observation (outpatient ED visit with an associated observation claim), transferred to another hospital (outpatient visit with a disposition recorded as transferred to another hospital), died in the ED (outpatient ED visit with a same day death). All other visits were recorded as having a disposition of discharged from the ED. [b]Beneficiaries age 65 and older seeking care in the emergency department at an acute care hospital in the 50 United States and the District of Columbia. [c]Visits in December of 2016 were excluded because we did not have complete 30-day cost data.

© 2020 Burke LG et al. *JAMA Network Open*.

**eTable 2. Adjusted[a] Rates of Disposition[b] From the Emergency Department (ED) by Year Among Medicare Beneficiaries[c] From 2011 to 2016**

| | Adjusted Mean 2011 | Adjusted Mean 2016 | Time Trend ($/year) | P-Value |
|---|---|---|---|---|
| **Discharged** | 59.5% | 61.9% | 0.50% (0.48% to 0.51%) | <0.001 |
| **Admitted/Transferred** | 33.8% | 28.9% | -0.98% (-0.99% to -0.97%) | <0.001 |
| **Observation** | 6.5% | 9.1% | 0.48% (0.477% to 0.493%) | <0.001 |
| **Died in the ED** | 0.099% | 0.078% | -0.003% (-0.004% to -0.002%) | <0.001 |

[a] Separate linear probability models were run for each disposition category above with disposition as the outcome, year as the predictor. The model incorporated hospital random effects and adjusted for primary visit diagnosis as well as beneficiary age, sex, Medicaid eligibility, race and chronic conditions as covariates. [b]A mutually exclusive hierarchy of disposition categories was created as follows: admitted (inpatient claim associated with the ED visit), observation (outpatient ED visit with an associated observation claim), transferred to another hospital (outpatient visit with a disposition recorded as transferred to another hospital), died in the ED (outpatient ED visit with a same day death). All other visits were recorded as having a disposition of discharged from the ED. [c] Beneficiaries age 65 and older seeking care in the emergency department at an acute care hospital in the 50 United States and the District of Columbia. [d] Visits in December of 2016 were excluded because we did not have complete 30-day cost data.

© 2020 Burke LG et al. *JAMA Network Open*.

**eTable 3. Unadjusted Total 30-Day Costs and Components of Spending by Year for Emergency Department (ED) Visits Among Medicare Beneficiaries From 2011 to 2016 Without and With Adjustment for Inflation**

| Unadjusted | | | | | | |
|---|---|---|---|---|---|---|
| | **2011** | **2012** | **2013** | **2014** | **2015** | **2016** |
| **Total 30 Day Costs**[a] | $8,717 | $8,301 | $8,492 | $8,355 | $8,446 | $8,942 |
| **Index ED Visit**[b] | $3,254 | $3,128 | $3,225 | $3,120 | $3,111 | $3,245 |
| **Physician Costs**[c] | $1,179 | $1,174 | $1,198 | $1,125 | $1,145 | $1,212 |
| **Total Post Index Spending** | $4,284 | $3,999 | $4,068 | $4,110 | $4,190 | $4,485 |
| **Outpatient**[d] | $352 | $382 | $383 | $431 | $433 | $479 |
| **Post-Acute Care**[e] | $2,320 | $2,094 | $2,139 | $2,164 | $2,235 | $2,258 |
| **Follow-up ED**[f] | $89 | $91 | $91 | $116 | $117 | $116 |
| **Follow-up Inpatient**[f] | $1,506 | $1,411 | $1,433 | $1,373 | $1,378 | $1,591 |
| **Follow-up Observation**[f] | $17 | $21 | $23 | $26 | $27 | $41 |
| | | | | | | |
| **Converted to 2016 Dollars (Using Consumer Price Index)** | | | | | | |
| | **2011** | **2012** | **2013** | **2014** | **2015** | **2016** |
| **Total 30 Day Costs**[a] | $9,301 | $8,677 | $8,749 | $8,471 | $8,553 | $8,942 |
| **Index ED Visit**[b] | $3,472 | $3,270 | $3,323 | $3,163 | $3,151 | $3,245 |
| **Physician Costs**[c] | $1,258 | $1,227 | $1,235 | $1,141 | $1,159 | $1,212 |
| **Total Post Index Spending** | $4,571 | $4,180 | $4,192 | $4,167 | $4,243 | $4,485 |
| **Outpatient**[d] | $375 | $400 | $394 | $437 | $438 | $479 |
| **Post-Acute Care**[e] | $2,476 | $2,189 | $2,204 | $2,194 | $2,263 | $2,258 |
| **Follow-up ED**[f] | $95 | $95 | $94 | $117 | $119 | $116 |
| **Follow-up Inpatient**[f] | $1,607 | $1,475 | $1,476 | $1,392 | $1,396 | $1,591 |
| **Follow-up Observation**[f] | $19 | $22 | $24 | $26 | $27 | $41 |

[a] Total standardized cost for all claims within 30 days of presentation to the ED for the index visit. [b]For admitted patients, this represents the cost of the associated hospitalization since there is no separate ED facility payment. For visits with an observation claim the index cost includes the observation claim in addition to the outpatient ED visit claim. For all other outpatient visits (discharged, transferred), the index cost represents the outpatient facility payment. [c] Physician costs include all physician claims in the 30-day period. Post-index costs include all other non-physician claims in the 30-day period. [d] Spending on all other non-ED outpatient care. [e] Aggregate spending on the following services: home health, hospice, skilled nursing facilities and durable medical equipment. [f] Spending on any spending on ED, inpatient or observation care, respectively.

© 2020 Burke LG et al. *JAMA Network Open.*

## eTable 4. Time Trends in 30-Day Costs Associated With Emergency Department Visits From 2011 to 2016 Among Medicare Beneficiaries by Adjustment Model

| | Unadjusted[a] | Adjusting for patient characteristics and diagnosis [b] | Adjusting for beneficiary chronic conditions[c] |
|---|---|---|---|
| **Total 30-Day Costs** | -$99 (-$104 to -$94) | -$86 (-$91 to -$82) | -$126 (-$130 to -$121) |
| **Index Visit Costs[d]** | -$47 (-$49 to -$45) | -$43 (-$44 to -$41) | -$48 (-$50 to -$47) |
| **Physician Costs[e]** | -$20.4 (-$21.0 to -19.9) | -$19.9 (-$20.4 to -$19.3) | -$25 (-$25 to -$24) |
| **Total Post Index Costs[f]** | -$32 (-$36 to -$28) | -$24 (-$28 to -$20) | -$53 (-$56 to -$49) |
| **Outpatient** | $16 ($14 to $19) | $16 ($14 to $19) | $15 ($12 to $18) |
| **Post-acute Care** | -$36 (-$37 to -$34) | -$29 (-$31 to -$28) | -$42 (-$44 to -$41) |
| **ED** | $5.0 ($4.8 to $5.2) | $5.1 ($4.9 to $5.3) | $4.6 ($4.4 to $4.8) |
| **Inpatient** | -$21 (-$23 to -$20) | -$20 (-$22 to -$18) | -$34 (-$36 to -$32) |
| **Observation** | $3.6 ($3.5 to $3.7) | $3.7 ($3.6 to $3.8) | $3.6 ($3.5 to $3.7) |

[a] Time trends were determined linear regression model with total 30-day costs (converted to 2016 dollars) and time (year) as the predictor and adjusting for hospital random effects (Model 1) in the initial model. [b]Model 2 further adjusts for principal diagnosis as well as beneficiary age, sex, Medicaid eligibility, race. [c]Model 3 further incorporates beneficiary chronic conditions (chronic conditions warehouse). [d]For admitted patients, this represents the cost of the associated hospitalization since there is no separate ED facility payment. For visits with an observation claim the index cost includes the observation claim in addition to the outpatient ED visit claim. For all other outpatient visits (discharged, transferred), the index cost represents the outpatient facility payment. [e]Physician costs include all physician claims in the 30-day period. [f] Post-index cost includes all other non-physician claims in the 30-day period. All results were significant at p<.001.

© 2020 Burke LG et al. *JAMA Network Open.*

## eTable 5. Adjusted[a] Total 30-Day Costs and Components of Spending by Year for Emergency Department (ED) Visits Among Medicare Beneficiaries From 2011 to 2016

| | 2011 | 2012 | 2013 | 2014 | 2015 | 2016 |
|---|---|---|---|---|---|---|
| **Total 30 Day Costs** | $8,851 | $8,341 | $8,379 | $8,161 | $8,136 | $8,143 |
| **Index ED Visit[b]** | $2,725 | $2,568 | $2,606 | $2,465 | $2,453 | $2,486 |
| **Physician Costs[c]** | $1,253 | $1,228 | $1,233 | $1,144 | $1,154 | $1,144 |
| **Total Post Index Spending** | $4,865 | $4,536 | $4,532 | $4,544 | $4,521 | $4,504 |
| **Outpatient[d]** | $462 | $487 | $480 | $525 | $528 | $534 |
| **Post-Acute Care[e]** | $2,520 | $2,276 | $2,283 | $2,299 | $2,292 | $2,210 |
| **Follow-up ED[f]** | $105 | $105 | $104 | $128 | $128 | $119 |
| **Follow-up Inpatient[f]** | $1,711 | $1,598 | $1,593 | $1,518 | $1,498 | $1,554 |
| **Follow-up Observation[f]** | $19 | $22 | $25 | $27 | $28 | $41 |

[a] Costs were converted to 2016 dollars using the Consumer Price Index. Adjusted rates derived from a linear regression model with total 30 day costs and time (year) as the predictor, adjusting for hospital random effects, principal diagnosis as well as beneficiary age, sex, Medicaid eligibility, race and chronic conditions. [b]For admitted patients, this represents the cost of the associated hospitalization since there is no separate ED facility payment. For visits with an observation claim the index cost includes the observation claim in addition to the outpatient ED visit claim. For all other outpatient visits (discharged, transferred), the index cost represents the outpatient facility payment. [c] Physician costs include all physician claims in the 30-day period. Post-index costs includes all other non-physician claims in the 30-day period. [d] Spending on all other non-ED outpatient care. [e] Aggregate spending on the following services: home health, hospice, skilled nursing facilities and durable medical equipment. [f] Spending on any spending on ED, inpatient or observation care respectively after the index ED visit.

© 2020 Burke LG et al. *JAMA Network Open*.

**eTable 6. Trends in Adjusted[a] Cost of the Index Visit[b] From 2011 to 2016 Among Medicare Beneficiaries Using the Emergency Department, Overall and Stratified by Disposition[c]**

| | Adjusted Mean 2011 | Adjusted Mean 2016 | Time Trend ($/year) | P-Value |
|---|---|---|---|---|
| **All Visits** | $2725 | $2,486 | -$48 (-$50 to -$47) | <0.001 |
| **Admitted** | $7,639 | $7,726 | $10 ($6 to $14) | <0.001 |
| **Transferred** | $929 | $957 | $4 ($2 to $6) | <0.001 |
| **Observation** | $1,726 | $2,100 | $87 ($86 to $88) | <0.001 |
| **Discharged** | $443 | $456 | $1.3 ($1.1 to $1.4) | <0.001 |

[a] Data are adjusted for inflation, hospital random effects, principal diagnosis as well as beneficiary demographics and comorbid conditions. [b] All facility costs associated with the index ED visit (including any associated inpatient or observation costs if the patient remained in the hospital for further care. [c]Beneficiaries were assigned a disposition from the ED according the following mutually exclusive hierarchy of categories, admitted (inpatient ED visit), observation (outpatient ED visit with an associated observation claim), died in the ED (outpatient ED visit with a same day death date), discharged from the ED.

© 2020 Burke LG et al. *JAMA Network Open.*

## eTable 7. Adjusted[a] Total 30-Day Costs and Components of Spending by Year for Emergency Department (ED) Visits Among Medicare Beneficiaries From 2011 to 2016 for the 10 Most Frequent Conditions[b]

| Chest pain | Slope (95% CI) | Syncope | Slope (95% CI) |
|---|---|---|---|
| Index Visit Costs[c] | -$42 (-$48 to -$36) | Index Visit Costs[c] | -$89 (-$97, -$81) |
| Physician Costs[d] | -$29 (-$31 to -$36) | Physician Costs[d] | -$12 (-$15, -$9) |
| Total Post Index Costs[e] | -$42 (-$49 to -$34) | Total Post Index Costs [e] | -$68 (-$81, -$56) |
| Outpatient [f] | $21 ($19 to $23) | Outpatient [f] | $19 ($16, $21) |
| Post-acute Care[g] | -$40 (-$44 to -$36) | Post-acute Care [g] | -$75 (-$83, -$66) |
| ED [h] | $7 ($6 to $8) | ED [h] | $6 ($5, $7) |
| Inpatient [h] | -$37 (-$43 to -$30) | Inpatient [h] | -$24 (-$32, -$15) |
| Observation [h] | $7 ($7 to $8) | Observation [h] | $6 ($5, $7) |
| | | | |
| Other Lower Respiratory Tract Disease | Slope (95% CI) | Pneumonia | Slope (95% CI) |
| Index Visit Costs[c] | $5 (-$4 to $14) | Index Visit Costs[c] | -$187 (-$198, -$175) |
| Physician Costs[d] | -$24 (-$26 to -$21) | Physician Costs[d] | -$13 (-$16 to -$9) |
| Total Post Index Costs [e] | -$30 (-$42 to -$18) | Total Post Index Costs [e] | -$82 (-$99 to -$65) |
| Outpatient [f] | $21 ($19 to $23) | Outpatient [f] | $19 ($16 to $22) |
| Post-acute Care[g] | -$19 (-$26 to -$13) | Post-acute Care [g] | -$55 (-$66 to -$44) |
| ED [h] | $4 ($3 to $4) | ED [h] | $3 ($2, $4) |
| Inpatient [h] | -$40 (-$49 to -$30) | Inpatient [h] | -$51 (-$63 to -$39) |
| Observation [h] | $4 ($3 to $4) | Observation [h] | $3 ($2, $3) |
| | | | |
| Abdominal pain | Slope (95% CI) | Chronic Obstructive Pulmonary Disease | Slope (95% CI) |
| Index Visit Costs[c] | -$77 (-$86 to -$67) | Index Visit Costs[c] | -$92 (-$98, -$85) |
| Physician Costs[d] | -$31 (-$34 to -$29) | Physician Costs[d] | -$16 (-$19, -$14) |
| Total Post Index Costs[e] | -$54 (-$65 to -$43) | Total Post Index Costs [e] | -$73 (-$86, -$60) |
| Outpatient [f] | $15 ($13 to $17) | Outpatient [f] | $15 ($13, $16) |
| Post-acute Care[g] | -$31 (-$37 to -$25) | Post-acute Care [g] | -$48(-$55, -$41) |
| ED [h] | $6 ($5 to $7) | ED [h] | $4 ($3, $5) |
| Inpatient [h] | -$46 (-$55 to -$38) | Inpatient [h] | -$48 (-$59, -$38) |
| Observation [h] | $3 ($2 to $3) | Observation [h] | $5 ($4, $5) |

© 2020 Burke LG et al. *JAMA Network Open.*

| Superficial Injury | Slope (95% CI) |
|---|---|
| Index Visit Costs[c] | $3 ($1 to $5) |
| Physician Costs[d] | -$16 (-$17 to -$14) |
| Total Post Index Costs[e] | -$32 (-$41 to -$23) |
| Outpatient [f] | $8 ($6 to $10) |
| Post-acute Care[g] | -$27 (-$32 to -$22) |
| ED [h] | $3 ($2 to $4) |
| Inpatient [h] | -$17 (-$24 to -$11) |
| Observation [h] | $2.1 ($1.7 to $2.4) |

| Cardiac Dysrhythmias | Slope (95% CI) |
|---|---|
| Index Visit Costs[c] | -$71 (-$81 to -$62) |
| Physician Costs[d] | -$25 (-$28 to -$22) |
| Total Post Index Costs [e] | -$26 (-$38 to -$13) |
| Outpatient [f] | $35 ($32 to $39) |
| Post-acute Care [g] | -$44 (-$51 to -$37) |
| ED [h] | $11 ($9 to $12) |
| Inpatient [h] | -$34 (-$43 to -$24) |
| Observation [h] | $6 ($5 to $7) |

| Urinary Tract Infection | Slope (95% CI) |
|---|---|
| Index Visit Costs[c] | -$103 (-$108, -$98) |
| Physician Costs[d] | -$20 (-$22, -$18) |
| Total Post Index Costs[e] | -$123 (-$134, -$112) |
| Outpatient [f] | $12 ($10, $14) |
| Post-acute Care[g] | -$97 (-$105, -$89) |
| ED [h] | $6 ($5 $6) |
| Inpatient [h] | -$46 (-$53, -$38) |
| Observation [h] | $3 ($3, $4) |

| Back Pain | Slope (95% CI) |
|---|---|
| Index Visit Costs[c] | -$14 (-$21 to -$7) |
| Physician Costs[d] | -$41 (-$44 to -$38) |
| Total Post Index Costs[e] | -$71 (-$85 to -$57) |
| Outpatient [f] | $12 ($10 to $15) |
| Post-acute Care [g] | -$53 (-$61 to -$45) |
| ED [h] | $2 ($0.4 to $3) |
| Inpatient [h] | -$36 (-$46 to -$25) |
| Observation [h] | $4 ($3 to $4) |

a Costs were converted to 2016 dollars using the Consumer Price Index. Adjusted rates derived from a linear regression model with total 30-day costs and time (year) as the predictor, adjusting for hospital random effects, principal diagnosis as well as beneficiary age, sex, Medicaid eligibility, race and chronic conditions. b Principal diagnosis category, defined by classified by Healthcare Utilization Project Single-Level Clinical Classifications Software Categories. [c]For admitted patients, this represents the cost of the associated hospitalization since there is no separate ED facility payment. For visits with an observation claim the index cost includes the observation claim in addition to the outpatient ED visit claim. For all other outpatient visits (discharged, transferred), the index cost represents the outpatient facility payment.
[d]Physician costs include all physician claims in the 30-day period. Post-index costs includes all other non-physician claims in the 30-day period. [e] All spending after the index ED visit (excluding physician professional claims). [f] Spending on all other non-ED outpatient care. [g] Aggregate spending on the following services: home health, hospice, skilled nursing facilities and durable medical equipment. [h] Spending on any spending on ED, inpatient or observation care respectively that occurred after the index ED visit (regardless of disposition at the time of the index visit).

© 2020 Burke LG et al. *JAMA Network Open.*

## eTable 8. Comparing Time Trends in 30-Day Standardized Costs Overall and by Spending Component, Adjusted for Hospital Fixed Effects vs Random Effects[a]

| | Adjusting for hospital random effects[b] | Adjusting for hospital fixed effects[c] |
|---|---|---|
| **Total 30-Day Costs** | -$125.71 (-$130.09 to -$121.34) | -$125.70 (-$130.08 to -$121.32) |
| **Index Visit Costs[d]** | -$48.49 (-$50.16 to -$46.83) | -$48.46 (-$50.12 to -$46.79) |
| **Physician Costs[e]** | -$24.58 (-$25.13 to -$24.04) | -$24.58 (-$25.12 to -$24.03) |
| **Total Post Index Costs[f]** | -$52.62 (-$56.37 to -$48.88) | -$52.66 (-$56.41 to -$48.92) |
| **Outpatient** | $15.11 ($12.31 to $17.91) | $15.10 ($12.29 to $17.90) |
| **Post-acute Care** | -$42.12 (-$43.7 to -$40.5) | -$42.15 (-$43.74 to -$40.55) |
| **ED** | $4.62 ($4.40 to $4.84) | $4.60 ($4.38 to $4.82) |
| **Inpatient** | -$33.81 (-$35.7 to -$31.9) | -$33.83 (-$35.7 to -$32.0) |
| **Observation** | $3.62 ($3.50 to $3.74) | $3.61 ($3.49 to $3.74) |

[a]Time trends were determined linear regression model with total 30-day costs (converted to 2016 dollars) and time (year) as the predictor adjusting for principal diagnosis as well as beneficiary age, sex, Medicaid eligibility, race. [b]The primary model incorporates hospital random effects. [c] A sensitivity analysis incorporates hospital fixed effects rather than random effects. [d]For admitted patients, this represents the cost of the associated hospitalization since there is no separate ED facility payment. For visits with an observation claim the index cost includes the observation claim in addition to the outpatient ED visit claim. For all other outpatient visits (discharged, transferred), the index cost represents the outpatient facility payment. [e]Physician costs include all physician claims in the 30-day period. [f] Post-index cost includes all other non-physician claims in the 30-day period.

© 2020 Burke LG et al. *JAMA Network Open.*

## eTable 9. Adjusted Time Trends[a] in Total 30-Day Costs and Components of Spending by Year for Emergency Department (ED) Visits Among Medicare Beneficiaries From 2009 to 2016

| | Slope | 2009 | 2010 | 2011 | 2012 | 2013 | 2014 | 2015 | 2016 |
|---|---|---|---|---|---|---|---|---|---|
| **Total Costs[b]** | -$267 (-$271 to -$264) | $9,795 | $9,998 | $9,001 | $8,474 | $8,396 | $8,078 | $7,955 | $7,865 |
| **Index Visit [c]** | -$99 (-$100 to -$97) | $3,102 | $3,039 | $2,836 | $2,660 | $2,655 | $2,476 | $2,410 | $2,407 |
| **Physician[d]** | -$35.8 (-$36.2 to -$35.4) | $1,377 | $1,345 | $1,261 | $1,236 | $1,229 | $1,129 | $1,135 | $1,115 |
| **Total Post Index[e]** | -$133 (-$136 to -$130) | $5,309 | $5,607 | $4,897 | $4,570 | $4,504 | $4,465 | $4,402 | $4,335 |
| **Outpatient** | $12 ($10 to $14) | $451 | $465 | $462 | $486 | $477 | $519 | $519 | $522 |
| **Post-acute Care** | -$76 (-$77 to -$75) | $2,606 | $3,010 | $2,499 | $2,261 | $2,246 | $2,245 | $2,225 | $2,132 |
| **ED** | $4.6 ($4.4 to $4.8) | $97 | $96 | $104 | $104 | $103 | $126 | $128 | $117 |
| **Inpatient** | -$77 (-$79 to -$76) | $2,100 | $1,981 | $1,773 | $1,656 | $1,614 | $1,507 | $1,462 | $1,483 |

[a]Time trends were determined linear regression model with total 30-day costs (converted to 2016 dollars) and time (year) as the predictor and adjusting for hospital random effects, principal diagnosis, beneficiary age, sex, Medicaid eligibility, race and chronic conditions using Centers for Medicare and Medicaid services Hierarchical Condition Categories (HCC). We used HCC in this analysis because we had this data available for all years, unlike the Chronic Conditions Warehouse categories that we had for only 2011-2016. [b] Total spending within 30 days of ED presentation for all visits. [c]For admitted patients, this represents the cost of the associated hospitalization since there is no separate ED facility payment. For visits with an observation claim the index cost includes the observation claim in addition to the outpatient ED visit claim. For all other outpatient visits (discharged, transferred), the index cost represents the outpatient facility payment. [d]Physician costs include all physician claims in the 30-day period. [e]Post-index cost includes all other non-physician claims in the 30-day period.

© 2020 Burke LG et al. *JAMA Network Open.*

## eTable 10. Trends in Adjusted Total 90-Day Costs[a] and Components of Spending for Emergency Department (ED) Visits Among Medicare Beneficiaries From 2011 to 2016

| | Adjusted Mean 2011 | Adjusted Mean 2016 | Time Trend ($/year) |
|---|---|---|---|
| **Total 90-Day Costs** | $14,346 | $13,266 | -$205 (-$216 to -$194) |
| **Index Visit Costs[b]** | $2,725 | $2,486 | -$48 (-$50 to -$47) |
| **Physician Costs[c]** | $2,377 | $2,168 | -$50 (-$51 to -$49) |
| **Total Post Index Costs[d]** | $9,240 | $8,596 | -$106 (-$117 to -$96) |
| **Outpatient[e]** | $1,086 | $1,284 | $35 ($26 to $45) |
| **Post-acute Care[f]** | $4,320 | $3,718 | -$87 (-$90 to -$85) |
| **ED[g]** | $239 | $261 | $11 ($11 to $12) |
| **Inpatient[g]** | $3,500 | $3,188 | -$74 (-$77 to -$71) |
| **Observation[g]** | $41 | $93 | $8.1 ($7.9 to $8.3) |

[a] Costs were converted to 2016 dollars using the Consumer Price Index. Adjusted rates derived from a linear regression model with total 90-day costs and time (year) as the predictor, adjusting for hospital random effects, principal diagnosis as well as beneficiary age, sex, Medicaid eligibility, race and chronic conditions. [b]For admitted patients, this represents the cost of the associated hospitalization since there is no separate ED facility payment. For visits with an observation claim the index cost includes the observation claim in addition to the outpatient ED visit claim. For all other outpatient visits (discharged, transferred), the index cost represents the outpatient facility payment. [c] Physician costs include all physician claims in the 30-day period. Post-index costs includes all other non-physician claims in the 30-day period. [d] Spending on all non-physician services after the index ED visit and associated inpatient/observation stay. [e]Spending on all other non-ED outpatient care. [f] Aggregate spending on the following services: home health, hospice, skilled nursing facilities and durable medical equipment. [g] Spending on any subsequent ED, inpatient or observation care respectively.

© 2020 Burke LG et al. *JAMA Network Open.*
